# Academic Self-Concept Wins the Race: The Prediction of Achievements in Three Major School Subjects by Five Subject-Specific Self-Related Variables

**DOI:** 10.3390/bs14010040

**Published:** 2024-01-05

**Authors:** Detlef H. Rost, Xiaoli Feng

**Affiliations:** 1 Center for Mental Health Education, School of Psychology, Southwest University Chongqing, 2 Tiansheng Road, Beibei, Chongqing 400715, China; 2 Faculty of Psychology, Philipps-University Marburg, 35032 Marburg, Germany; 3 Research Center for Modern Linguistics and Foreign Language Education, College of International Studies, Southwest University Chongqing, 2 Tiansheng Road, Beibei, Chongqing 400715, China; fengxiaoli168@126.com

**Keywords:** academic self-concept, conscientiousness, need for cognition, perseverance of effort, consistency of interest, academic achievement, school subjects, adolescents, regression analysis

## Abstract

The importance of self-related constructs in predicting academic achievement has been increasingly emphasized in recent decades. Typically, bivariate associations of self-related variables with achievements have been reported. Research quantifying the combined predictive power of more than two self-variables has been scarce. Moreover, except for the academic self-concept, these variables have almost always been measured across domains, i.e., without considering the specifics of individual school subjects. The current study aimed to statistically predict academic achievement (operationalized via school grades) in three major subjects (Chinese (native language), mathematics, and English (foreign language)) by using subject-tied scales, namely academic self-concept, conscientiousness, need for cognition, perseverance of effort, and consistency of interest. The sample comprised 791 Chinese adolescents. Each scale was related separately to each of the three school subjects. Hierarchical linear regression analyses were run. The control variable, biological sex, accounted for 2% of Chinese grades and 8% of English grades, but not of mathematics grades. Adding subject-specific self-concept scales increased the explained variance to 7% (Chinese), 16% (mathematics), and 32% (English). Further additions to the other four self-related scales did not increase the variances that were accounted for. The discussion underlines the relevance of subject-specific academic self-concepts as predictors for subject-tied academic achievements.

## 1. Introduction

In addition to cognitive abilities, self-related variables have been discussed as important determinants of (academic) achievement for the last three decades. Morin refers to “self” as an umbrella term that comprises “all imaginable physical and psychological (e.g., cognitive, affective, motivational, social) characteristics that make a person unique and different from others” [1] (p. 3). Among the Big Five personality traits, conscientiousness is more closely related to academic achievement than agreeableness, extraversion, openness, or neuroticism [2,3,4,5]. Conscientiousness is a “robust predictor of student performance” and “accounts for performance above and beyond cognitive ability” [6] (p. 245). However, motivational variables are generally considered better predictors than general personality traits [7]. Against this background, the present study investigated the factorial structures and psychometric properties of five self-related school subject-specific scales (hereafter in short: self-scales), namely academic self-concept, conscientiousness, need for cognition, perseverance of effort, and consistency of interest. Each scale was administered in three versions, each addressing one of the three main school subjects (native language Chinese, mathematics, and foreign language English). Subsequently, their intercorrelations and relationships with academic achievement were calculated for the three school subjects. Hierarchical linear regression analyses were subsequently used to quantify the joint power of these five self scales in predicting school grades. In addition, the incremental validity of each of the five variables above and beyond the other four variables was quantified.

These five variables were selected for the following two reasons: first, they are among the most frequently discussed non-cognitive determinants of scholastic performance, and second, most of the relevant primary studies have only reported bivariate correlations with (academic) achievement but have failed to analyze the combined predictive power of these variables for academic achievement.

### 1.1. Self-Related Constructs and Achievement

#### 1.1.1. Academic Self-Concept (ASC)

The self-concept comprises the knowledge, beliefs, and attitudes of a person about himself or herself. In other words, it is “a person’s mental model of his or her abilities and characteristics” [8] (p. 750) and predicts a variety of behaviors in everyday life [9].

An early meta-analysis that was conducted by Hansford and Hattie [10] revealed an average correlation of *r*_Ø_ = 0.18 between overall (domain-general) self-concept and achievement, whereas self-concept of ability was more closely associated with achievement (*r*_Ø_ = 0.42). The ASC, i.e., the way a person describes and evaluates his or her academic performance, is one of the most important motivational factors in education [11,12,13,14,15,16,17,18,19,20,21]. Until the 1970s, the ASC was often defined and measured without taking into account the differences between school subjects. However, students’ different experiences in diverse school subjects lead to different perceptions of educational settings and scholastic successes and thus to the development of a multidimensional ASC (i.e., consisting of different factors). It has now been widely agreed that there are many ASCs [22,23,24], as “students align their self-concepts closely to the curriculum they encounter” [25] (p. 491). As school experience increases, the domain specificity of the ASC increases [26,27], and a reciprocal relationship with performance develops [28,29,30,31,32,33,34]. Today, most operationalizations of the ASC are contextual, referring directly to single school subjects [12,13,22,30,35,36,37,38,39,40,41,42,43]. There is ample evidence that performance in specific school subjects (mathematics and language subjects have been studied most often) can be well predicted via their associated (i.e., subject specific) ASCs [12,13,22,30,35,36,37,38,39,40,41,42,43,44,45,46,47,48,49], even across different performance levels and even after controlling for intelligence and previous scholastic achievement [16,50,51]. As with many other motivational variables, ASCs are more strongly linked to the grades given by the teacher than to the results of standardized achievement tests [19,52]. ASCs in mathematics and German were found to be good predictors of matched grades (*r* = 0.69 and *r* = 0.48, respectively) and even outperformed intelligence (*r* = 0.33 and *r* = 0.28, respectively) [53]. A meta-analysis [19] reported averaged correlations of verbal and mathematic self-concepts with corresponding achievements of *r*_Ø_ = 0.35 and *r*_Ø_ = 0.43, respectively. Huang’s analytical review [28] yielded slightly lower coefficients for longitudinal predictions of academic achievement via subject-specific ASCs.

In addition, it has been found that subject-linked ASCs are more closely associated with assigned academic performance than a global self-score or a cross-subject general ASC [30,46,54]. These findings were confirmed with a series of studies that examined the relationships of six subject-specific ASCs (biology, English, German, history, mathematics, and physics) with adolescents’ scholastic achievement in eleven samples [39]. The medians of the general (i.e., cross domain) ASC achievement correlations ranged from *r*_Md_ = 0.33 (biology) to *r*_Md_ = 0.41 (history). In contrast, the lowest median of the subject-matched (convergent) correlations was *r*_Md_ = 0.49 (biology), and the maximal was *r*_Md_ = 0.62 (mathematics). The medians of the divergent coefficients (ASCs and grades addressed to different subjects) were markedly lower, varying from *r*_Md_ = 0.10 for the English ASC to *r*_Md_ = 0.23 for the History ASC. A meta-analysis that was conducted by Möller et al. [19] revealed similar results for the relationships between mathematics and verbal self-concepts and mathematics and verbal achievements. The Trends in International Mathematics and Science Study 2019 [55] has once again proven that it is particularly useful to focus ASCs and academic achievements on the same topic. In large samples from Japan, Norway, and the United States, the science-specific self-concept emerged as “the strongest predictor of science achievement among motivational constructs” [56] (p. 22).

At this point, it should be noted that academic self-efficacy (ASE) is also a strongly performance-related construct [57,58,59]. Both of these self-variables, ASC and ASE, are strongly correlated with each other and with academic performance, but they are not interchangeable (i.e., identical) constructs that are merely labeled differently. They are anchored in distinct psychological theories, can be separated through factor analytical methods, refer to different frames of reference, develop differently, and differ in their unique power to predict academic performance [59,60]. ASE is almost exclusively defined in domain-specific terms. It therefore generally predicts domain-specific performance better than a cross-domain construct (such as a general ASC). However, if ASC and ASE are measured at the same aggregate level (both are tied to the same subject), then subject-specific ASCs are equivalent or even superior to subject-specific ASEs in predicting students’ subject-specific academic achievements [12].

#### 1.1.2. Conscientiousness (CSN)

Of the Big Five personality dimensions, the content-unspecific (general) trait CSN displays the strongest relationship with academic performance [61,62,63,64]. Individuals scoring higher on CSN questionnaires are more responsible, reliable, and hardworking than those with lower scores. They take their tasks seriously and carry them out carefully. As a result, conscientious students tend to perform better than less conscientious students [63,64]. A comprehensive review [64] and several meta-analyses [2,65,66] have summarized the relationship between CSN and academic performance at school and college. Their reported effects ranged from ρ = 0.17 to ρ = 0.28 and were largely independent of intelligence [2]. A second-order meta-analysis confirmed these findings [67]. Across domains, the overall relationship between CSN and achievement tests was ρ = 0.22, and that between CSN and the grade point average (GPA) was ρ = 0.28. Meyer et al. [68] also reported stronger correlations with school grades than with standardized scholastic achievement tests (ρ = 0.28 vs. ρ = 0.13, respectively).

To what extent can CSN statistically predict academic achievement in conjunction with intelligence, the other four Big Five factors (openness, extraversion, agreeableness, and neuroticism), self discipline, and self efficacy? A hierarchical linear regression analysis by Dumfart and Neubauer [7] addressed this question. The dependent variables were GPA as well as grades in science and language subjects. Of the non-cognitive predictors, only CSN emerged as relevant after controlling for age, gender, and cognitive ability. The authors emphasized that CSN is “the crucial noncognitive predictor for school achievement” [7] (p. 14), thus substantiating previous research results [69,70]. The results of a recent regression analysis to predict the examination results of young British people confirmed this finding. Among several independent variables (adolescents’ intelligence, maternal cognitive ability, Big Five personality factors, and indicators of socioeconomic status), students’ intelligence was the best predictor of the General Certificate of Secondary Education (GCSE), followed by CSN. In contrast, the predictive power of each of the other variables was much lower [71]. This outcome was replicated with an extremely large sample of German adolescents [72].

#### 1.1.3. Need for Cognition (NFC)

Like for CSN, the NFC construct has been conceptualized as a stable personality trait that cuts across domains [73,74]. It refers to individuals’ general preference for mentally processing complex intellectual problems and solving them through cognitive effort. The NFC construct is not an ability construct but reflects an intrinsic cognitive motivation “to engage in and enjoy thinking” [73] (p. 116). This domain-general variable has often been measured with questionnaires developed by Cacioppo et al. [74] or modifications of their scales.

The NFC construct has been positively related to learning outcomes, final grade expectations in mathematics and language subjects, and general scholastic achievement (GPA) [75,76,77,78]. Meta-analyses have reported correlations of *r* = 0.17 and *r* = 0.26 between the NFC construct and academic achievement and between the NFC construct and college entrance test scores, respectively [2,79,80].

We only found one study that examined the extent to which the relationship between NFC and academic achievement is moderated via the particular ability-based learning environment. For secondary school students attending the highest (i.e., most demanding) track, the mean correlation between overall NFC and academic achievement in three majors was *r*_Ø_ = 0.18. For students attending the middle track (i.e., moderately demanding), it was *r*_Ø_ = 0.11. For students attending the low track (i.e., least demanding), it was *r*_Ø_ = 0.01 [81].

Psychometric test performances in French and German were exclusively predictable via subject-tied NFC (γ = −19 and γ = 0.08, respectively), but not by general NFC (both subjects: γ = 0.00). For mathematics, both its general and subject-tied NFC values were predictive, with the subject-specific factor having a significantly greater relevance [82].

#### 1.1.4. Perseverance of Effort (POE) and Consistency of Interest (COI)

The personality disposition “grit” refers to a person’s ability to pursue long-term goals with passion and persistence [83,84]. It has been conceptualized as a hierarchically structured “domain-general trait” [85] (p. 11), composed of two correlated primary factors, POE and COI, which are said to form the secondary factor grit. The POE construct encompasses the ability to sustain efforts for short- and long-term goals in the face of challenges, obstacles, and setbacks. The COI construct characterizes the ability to consistently focus on a goal or content over a long period of time. Based on the assumed hierarchical structure, Duckworth and colleagues suggested amalgamating POE and COI into one global (i.e., across domain) variable [83,84,85,86,87]. They claimed that (excellent) academic performance could be better predicted via grit than via intelligence [86,87], a statement that contradicts the results of decades of research showing that general intelligence *g* is by far the best single predictor of achievement [81,82,83,84,85,86,87,88,89,90,91,92,93,94,95,96]. Duckworth’s book on grit [86] and her grit scales [83,84] quickly gained a wide level of popularity [87,97,98,99] and were translated into many languages.

The global grit score has come under heavy fire. Criticisms have referred to its factorial structure, the lack of validity of the global grit score for achievement, and the alleged “novelty” of the construct. The hierarchical factor structure of grit that was postulated by Duckworth and colleagues [83,84] and other authors [100,101,102,103,104,105] cannot be conformed empirically, as the fit indexes of a hierarchical model with only two primary factors are always identical to those of a non-hierarchical model with two primary factors [106,107,108,109,110].

Moreover, a meta-analysis that was carried out by Credé et al. [106] found that merging the POE and COI constructs into one global grit score significantly decreased the predictive power of academic performance (GPA) compared to the predictive power of POE alone (ρ = 17 vs. ρ = 26, respectively). The COI construct was even less related to GPA (ρ = 10). Subsequent studies also showed that the POE construct was significantly more strongly correlated with academic achievement than the COI construct, although the magnitude of the correlation coefficients varied substantially across samples. Therefore, the global cross-domain grit construct, and thus its global score, was predominantly abandoned in favor of using the two primary factors, POE and COI, separately [111,112,113,114,115,116,117,118,119,120].

As far as the COI construct is concerned, a cross-domain individual interest contradicts the theory of interest. There has always been a consensus that a general academic interest, detached from specific content, does not exist [121,122,123,124,125]. In other words: “Individuals are not ‘interested’ in the same way that they may be ‘extraverted’… They are interested in something” [126] (p. 98). In the school setting, subjects moderate the relationship between interest and achievement. The only meta-analysis in this regard [127] reported average correlations between interest and achievement of *r*_Ø_ = 0.31, ranging from *r*_Ø_ = 0.17 for biology to *r*_Ø_ = 0.35 for science. In a large-scale study, the average correlation between interest and grades (five subjects: biology, chemistry, German, mathematics, and physics) was of a similar size [128].

Finally, it has been repeatedly shown that the POE construct overlaps extremely highly with the Big Five factor CSN [105,106,112,129,130]. This raises the question of whether the POE construct can be used to predict academic achievement beyond CSN.

### 1.2. Redundancies

The preceding remarks have shown that the five self-related constructs discussed are positively correlated with academic achievement. However, their variances overlap to a great extent in some cases, and they form a complex nomological network. A few examples of the many redundancies include the meta-analysis that was conducted by Credé et al. [106], which revealed strong associations of CSN with POE and COI (*r* = 0.83 and *r* = 0.61, respectively) and of POE with COI (*r* = 0.60). These high correlations were subsequently confirmed through several studies [111,112,120,130,131]. Medium-to-low correlations have been reported for the association between ASC with CSN and NFC, but only small correlations have been uncovered between NFC and CSN [14,75,132,133,134]. It has been shown that interests in different school subjects share many common elements with corresponding ASCs, up to a correlation of *r*_Ø_ = 0.77 [13,27,135,136,137].

### 1.3. Domain Specifities

Different school subjects are perceived differently by students and require different types of engagement [138,139]. Therefore, when interpreting the predictive powers of self-variables for academic achievement, it is useful to take subject-specific dependencies into account. This is because subject-specific measurements are usually more closely related to the corresponding subject-specific performance than are general (i.e., cross subject) measurements: they are symmetric concerning their degrees of generality. In other words, predictors and criteria should be operationalized under the same level of abstraction [9,140,141,142,143,144,145,146,147,148,149].

In the case of the ASC, its measurement has been predominantly linked to school subjects, often mathematics, science, and language subjects [26,30,37,38,42,49], but also to minor subjects such as sports and physical education [45,150,151], music [152,153,154,155], dancing [156], and arts [157]. However, for the other four self-concepts discussed so far, little or no subject-specific measurements have been performed to date.

Even with CSN, which has traditionally been conceptualized and measured as a cross-domain construct [73,74,158,159,160,161,162,163,164], it is not inconceivable, indeed likely, that it varies across school subjects. Everyday experiences have shown that a student may be diligent and conscientious in one subject but less industrious and more careless in another. However, we could not find any study that assessed CSN in a domain-specific way.

The COI construct has been designed as a trait-like characteristic that applies to all possible interests, regardless of a specific object. However, “there is no such thing as objectless interest” [165] (p. 137). Concerning scholastic interests, we have already noted that academic interests obviously and indisputably develop from a student’s engagement with subject-tied content. Thus, for many years, interest theory and interest research have contradicted the underlying assumption of the COI construct in that individual interests are object unspecific and can be validly measured across domains [13,121,122,123,124,125,126,127,128,166,167,168,169,170]. Such subject-linked relationships with academic achievement may also be relevant for the POE construct. Students’ propensity to stick with a subject and continue to strive despite setbacks and obstacles is likely to depend on the perceived difficulty of a school subject and on prior learning experiences, which may vary from subject to subject. For grit with POE and COI, this domain specificity was only addressed a few years ago, mainly concerning language subjects and mathematics [102,171,172,173,174,175,176,177], but also sports [178,179].

We only identified two papers that addressed a possible domain specificity of NFC. Pechtl [180] studied business students and concluded that NFC should be measured in a domain-specific manner, as is common for many school-related motivational and volitional variables. In line with this study, another study measured subject-specific NFC (four subjects) among high school students [82].

### 1.4. Recent Study

The ASC, CSN, NFC, PER, and COI constructs may be partially redundant in terms of their power to predict academic achievement. Therefore, the present study is the first in which these five self-variables were used simultaneously to jointly predict academic achievement in three main school subjects, namely Chinese, mathematics, and English.

To maximize their potential predictive power, the predictors and criteria should address the same subject. Therefore, the ASC was measured in a subject-specific manner. Unlike most of the previous studies, the other four predictors (CSN, NFC, POE, and COI) were also measured on a subject-specific basis. Thus, the Brunswick symmetry [144,145], i.e., the specificity-matching principle [9], was taken into account. This means that a cross-domain criterion (such as GPA) can be better predicted using a cross-domain (i.e., general) ASC than via a domain-specific one. On the other hand, in the case of a domain-specific criterion, such as a grade in a specific school subject, a subject-specific ASC can predict school performance better than a cross-subject (i.e., general) one.

The objective of the current study was to provide an answer to the following questions:

(1) Within each of the five self-variables (ASC, CSN, NFC, POE, and COI), is it possible to separate three main school subjects (Chinese, mathematics, and English) via factor analyses (with the self-variable held constant in each case)?
(2) Within each of the three main school subjects (Chinese, mathematics, and English), is it possible to separate the five self-variables (ASC, CSN, NFC, POE, and COI) via factor analyses (with the school subject held constant in each case)?
(3) What is the joint explanatory power of the five subject-specific self-scales (ASC, CSN, NFC, POE, and COI) in predicting the linked academic achievement in three main school subjects (Chinese, mathematics, and English)?
(4) What is the incremental (i.e., unique) validity of each of the five subject-specific self-variables (ASC, CSN, NFC, POE, and COI) in predicting their corresponding academic achievement in three main school subjects (Chinese, mathematics, and English) when the other four variables have been previously controlled? Or, to put it in another way, what is the extent to which each of the five variables, by itself and not in conjunction with the other predictors, statistically predicts school grades?

In the first step, we checked whether the assumed one-dimensionality of each of the fifteen subject-specific self-constructs (five variables × three subjects) was given. Based on these results, we formed 15 self-scales and calculated their psychometric properties (second step). In the third step, the intercorrelations of all the variables were established. In the fourth step, a series of hierarchical linear regression analyses were run to statistically predict the variance in school grades. Of particular interest was the amount of the subject-tied incremental validity of each self-scale after controlling for the remaining four.

## 2. Materials and Methods

### 2.1. Sample and Procedure

The initial sample comprised 807 adolescents from 20 middle school classes of 3 schools from a Chinese megacity. The smallest class comprised 30 students, and the largest comprised 47 pupils. A freely given informed consent was obtained from the parents, headmasters, and teachers. Parents and students were informed that their participation was completely voluntary, that all personal data would remain anonymous, and that non-participation would not result in any personal disadvantage. The survey took place in the classrooms during regular school hours and lasted less than 15 min. It was administered by an experienced psychologist. Six students did not want to participate. Due to not carefully filling the questionnaire in (16 questionnaires with missing responses or systematic ticking patterns could not be used), the statistical analyses of motivational variables were based on an effective sample of *N* = 791 (age: *M* = 15.76 years; *SD* = 0.78; boys: 48%). School grades were not available for 12 participants. Thus, analyses including academic achievement were based on *N* = 779. Once the file was completed, the assignment list (names associated with numbers) was given to the schools.

### 2.2. Variables

#### 2.2.1. Age, Sex, and Academic Achievement

Participants noted their age and sex. School grades for Chinese (native language), mathematics, and English (foreign language) were taken from the most recent school report card. Higher scores corresponded to better grades. Grades were chosen as performance criteria, as they are a central factor in the formation of students’ achievement-related self-cognition and determine their school career [181,182,183]. They are extremely significant for their future life trajectories and are real-life criteria. Therefore, they are ecologically valid by definition.

#### 2.2.2. Self-Variables

Five self-constructs, one personality variable (CSN), as well as four motivation variables (ASC, NFC, NFC, POE, and COI), were measured, each of which was specifically operationalized for three school subjects. The items, which focused on one of three school subjects (Chinese, mathematics, or English), were sequenced at random. The order was the same for all of the classes. Each item was presented in three versions: the wording of the stem was always identical. Only the subject specification (Chinese, mathematics, or English) differed.

**Figure 1:**
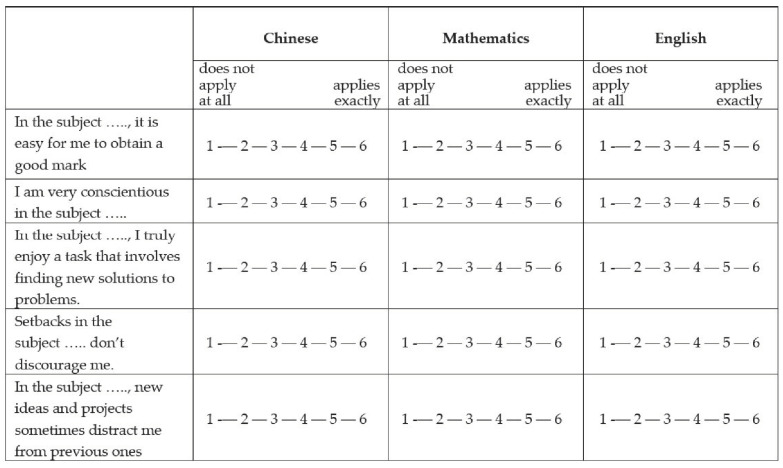
Layout of the questionnaire.

The questionnaire was designed in the form of a table (grid layout, see Figure 1). The first column contained one item per row. Each item had a placeholder (…) for the respective domain. The headline labels of the next three columns indicated the respective subject. In the cells (item × topic), students marked the extent to which each statement personally applied to them. The response options were identical for all of the items and ranged from “1 = does not apply at all” to “6 = applies exactly”. Thus, the same item stem was the basis for three items formulated in parallel, each relating to a different school subject. The usefulness of a grid design for the fast and efficient measurement of self-variables has been demonstrated in many studies [12,13,38,39,40,41,50,111,112,132,142,169,184,185,186,187].

To measure the ASC, CSN, POE, and COI variables, we used items that had already been shown to be appropriate for Chinese adolescents. [12,13,111,112]. The NFC items were translated into Chinese. With the help of two bilingual psychologists, the accuracy of the translations was checked via back-and-forth transcription [188,189].

The six ASC items were taken from the differential self-concept grid [39,40,190]. For an example, see the first item of Figure 1. CSN was measured with eight items from the Big Five Personality test (B5T) [164]. Figure 1, second item, shows an example. The six NFC items were adopted from Preckel [191]. An example is shown in Figure 1, third item. In addition, we administered the eight items of the short grit questionnaire [84]. However, in line with many factor analytic studies, no total grit score was formed in favor of the two separate self-scales POE (four items; example: Figure 1, fourth item) and COI (four items; example: Figure 1, fifth item). As the wording of the COI items reflects a lack of interest, their scores were reversed.

### 2.3. Statistical Data Treatment

Statistical analyses were performed using IBM SPSS 25 and IBM AMOS 25. All variables, except for age and biological sex, were class-wise z-standardized (M = 0.00; SD = 1.00). Such cluster-centered standardizations eliminate any intraclass correlations. Indeed, our study focused on individual effects, i.e., on level 1 associations [192]. Level 2 or level 3 correlations (class or school effects) were not of interest. Product-moment correlations were calculated. Correlations were averaged (*r*_Ø_) via Fisher’s *z*-transformation [193,194]. The sample was sufficiently large for the statistical methods that were applied [195,196,197,198,199,200,201]. Due to the large sample size, the significance level was set to 1% [202].

Confirmatory factor analyses (CFAs, maximum likelihood estimation) were run to test the appropriateness of a model with three subject-specific factors within each of the five self-constructs on the one hand and a model with five self-factors within each of the three subjects on the other hand. Correlated measurement errors were only accepted for items with identical wording stems (just the name of the school subject differed). For larger samples, the global fit index (χ^2^) usually reaches statistical significance whether a model fits the data or not. For this reason, and as different fit indexes respond differently to misspecification and model mismatches, we used the ratio χ^2^/df and a combination of three fit indexes, namely the incremental index CFI and the absolute indexes SRMR and RMSEA [203,204,205,206]. The frequently cited cut-off values for fit indexes recommended by Hu and Bentler [204] seem to be too strict for questionnaire data. Therefore, it has been recommended to apply less strict standards for questionnaire studies and to tolerate moderate deviations [207,208,209]. Hence, we applied the following simple cut-off criteria: RMSEA ≤ 0.08 and SRMR ≤ 0.08 (“badness-of-fit” indexes), and CFI ≥ 0.90 (“goodness-of-fit” index). We prioritized theoretical fit over adherence to the stated rules of thumb, as they “are rather arbitrary [and] should not be taken too seriously” [210] (p. 52). The accepted model should perform better in competition with a general factor model. For model comparison, we additionally used the entropy-based information criterion AIC [211,212,213]. A smaller AIC stands for a better fit.

Psychometric scale properties and variable intercorrelations were calculated. Subsequently, the relative relevance of each subject-specific self-scale for predicting its corresponding academic achievement was identified through running three hierarchical linear regression analyses, one for each criterion (Chinese grade, mathematics grade, and English grade). All regressions were structured in the same way. The two control variables sex and age were always entered first (step 1). Based on the hypothesis that among the measured constructs, the ASC is the best predictor of academic achievement, it was added as step 2. In step 3, the remaining four self-scales (CSN, NFC, POE, and COI) were added. Finally, we determined the unique contribution of each variable to the regression effect (i.e., the amount of the predicted variance not shared with the other four self-scales, step 4). To avoid interpretation problems due to high multicollinearity, the maximum predictor–predictor correlation was limited to *r* = 0.85, and no variance inflation factor (*VIF*) was allowed to exceed the limit of 3.5 [188,189].

## 3. Results

### 3.1. Factor Analyses

Table 1 shows the fit indexes of the measurement models when three distinguishable school subject factors were postulated within each of the five self-constructs, i.e., when the self-variable was always held constant. None of the five motivational constructs was adequately described by a general (i.e., content unspecific) factor. Instead, a structure with the subject factors Chinese, mathematics, and English fitted the data in each case. Each item marked “its” motivational factor according to theory. The averaged factor loadings ranged from λ_Ø_ = 0.51 (English COI) to λ_Ø_ = 0.78 (Chinese ASC).

The fit indexes of the models with five distinguishable self-factors within each of the three school subjects are shown in Table 2. In each case, the school subject was held constant. Unsurprisingly, a general factor model was again proven to be inconsistent with the data. The theoretically coherent models with five self-factors (ASC, CSN, NFC, POE, and COI) within each of the three school subjects had sufficient fit indexes. Again, each item indicated its respective self-factor. The averaged factor loadings ranged from λ_Ø_ = 0.51 (English COI) to λ_Ø_ = 0.75 (English ASC).

### 3.2. Psychometric Scale Properties

Based on the results of the CFAs, fifteen subject-specific self-scales were formed (three subjects × five self-constructs). All 15 scales were unimodally distributed. Their psychometric properties are listed in Table 3. Skewness and kurtosis values were well below ǀ1ǀ. Thus, univariate normality was not seriously violated. The internal consistencies of the ASC, CSN, and NFC scales proved to be satisfactory (ranging from α = 0.80 to α = 0.88). The POE scales declined in this respect (from α = 0.71 to α = 0.73). The internal consistencies of the COI scales were hardly sufficient (from α = 0.58 to α = 0.66). This was only partly due to their shortness, as the POE scales, each also only comprising four items, had significantly better internal consistencies. If the COI scales were extended by two equivalent items from four to six (i.e., on the length of the ASC and NFC scales), the internal consistencies would only increase to α = 0.74 (Chinese), α = 0.69 (mathematics), and α = 0.67 (English).

### 3.3. Intercorrelations

All correlations are presented in Table 4. Age did not correlate with any of the other variables. The variances of the five self-scales showed a high degree of overlap in many cases. For example, within each of the four self-scales, the averaged intercorrelations of the three subject-tied scales were quite high (COI: *r*_Ø_ = 0.70, CSN: *r*_Ø_ = 0.59, NFC: *r*_Ø_ = 0.53, and POE: *r*_Ø_ = 0.42), while the ASC scales were significantly less related to each other (*r*_Ø_ = 0.29). Mirroring this, four of the five self-sales (ASC, CSN, NFC, and POE) were strongly correlated within the same subject (Chinese: *r*_Ø_ = 0.65, mathematics: *r*_Ø_ = 0.68, and English: *r*_Ø_ = 0.70). However, COI made an exception with *r*_Ø_ = 0.22 (Chinese), *r*_Ø_ = 0.28 (mathematics), and *r*_Ø_ = 0.27 (English).

For the control variable biological sex (coding: 0 = boys and 1 = girls), there were statistically significant associations with grades in Chinese and English (*r* = 0.15 and *r* = 0.28, respectively). The highest correlation between a subject-tied scale and the corresponding grade was found for the English ASC (*r* = 0.53). The average association of the ASC scales with the matched grades was *r*_Ø_ = 0.39. The corresponding averaged coefficients for CSN, NFC, and POE were uniformly smaller (*r*_Ø_ = 0.23, *r*_Ø_ = 0.27, and *r*_Ø_ = 0.23, respectively). By far, the smallest mean correlation with grades was observed for COI (*r*_Ø_ = 0.10).

### 3.4. Statistical Prediction of Academic Achievement

Hierarchical linear multiple regression analyses were run to determine the predictive validity of the subject-tied self-scales in statistically explaining the corresponding school grades. There were no significant multicollinearities that could threaten the interpretation of the regression analyses (maximal predictor–predictor correlation *r* = 0.78; maximal *VIF* = 3.03). Table 5 shows the results of the regressions.

Of the two control variables biological sex and age, only the first (sex) was a statistically significant predictor in model 1 for Chinese (β = 0.15) and English (β = 0.38), but not for mathematics (β = −0.01). The variance that was accounted for amounted to 2% (Chinese) and 8% (English). Model 2 added the ASC, which was an additional statistically significant and relevant predictor for each grade (Chinese: β = 0.22; mathematics: β = 0.40; and English: β = 0.50). This second step accounted for 7% (Chinese), 16% (mathematics), and as much as 32% (English) of the achievement variance. The addition of CSN, NFC, POE, and COI (model 3) had little effect on the results. None of the four additional self-scales had a statistically significant β weight. The increment in explained variance was negligible for the English grade (∆*R*^2^ = 0.01), and the explained criterion variance did not increase at all for the Chinese grade and mathematics grade (∆*R*^2^ < 0.01). Hence, when calculating the unique (i.e., nonshared) significance of each scale in explaining the scores statistically, relevant increments (after controlling for the other four variables) were exclusively obtained from the ASCs. The increase was 5.0% for Chinese, 16% for mathematics, and as much as 24% for English.

## 4. Discussion

Based on a sample of Chinese adolescents, the present study aimed to shed light on the extent to which subject-specific academic achievements in three major school subjects (criteria) can be statistically predicted using five subject-tied self-constructs. The school subjects were Chinese, mathematics, and English. Two control variables (sex and age) and five subject-focusing self-variables served as predictors: academic self-concept (ASC), conscientiousness (CSN), need for cognition (NFC), perseverance of effort (POE), and consistency of interest (COI). The school grades from the last report card were used as ecologically valid operationalizations of subject-tied academic achievements (i.e., real-life criteria).

To the best of our knowledge, this study is the first to test the joint power of five subject-specific self-constructs for statistically predicting academic achievement in three school subjects. The questions that were posed at the beginning (1.4) of this study can be answered as follows:

(1) Within each of the five self-variables (ASC, CSN, NFC, POE, and COI), the three subjects (Chinese, mathematics, and English) were confirmed to be separate factors.
(2) Similarly, within each of the three school subjects (Chinese, mathematics, and English), the five self-variables (ASC, CSN, NFC, POE, and COI) were confirmed to be separate factors.
(3) Based on the results of the CFAs, fifteen approximately normally distributed scales (three subjects × five self-constructs) were formed, each with a good (ASC, CSN, and NFC), satisfactory (POE), or sufficient (COI) internal consistency. These self-scales served as predictors of achievement variance within each of the three school subjects. All five variables together statistically explained 7%, (Chinese), 16% (mathematics), and 33% (English) of the grade variances.
(4) Hierarchical linear multiple regression analyses evidenced that CSN, NFC, POE, and COI, in addition to the ASC, contributed virtually nothing independently to the statistical explanation of the subject-specific grade variances (the increments were only 1% in English and less than 1% in the other two subjects, Chinese and mathematics). In other words, the increments were minuscule and completely negligible in all three subjects. For the ASC, on the other hand, the proportion of explained variance in academic performance that was not shared with the other scales was substantial and varied by school subject (Chinese: 5%, mathematics: 16%, and English: 24%).

The results are clear and easy to grasp; therefore, the discussion can be brief. The bivariate correlations of the one-dimensional subject-specific self-scales ASC, CSN, and NFC with the assigned academic achievement are roughly within the range of what has been reported in the literature. However, we found extremely low correlations between the subject-linked interest scales (COI) and school grades in mathematics and English, which were clearly below the meta-analytic interest–achievement relationship of *r* = 0.31 [127]. For Chinese, we even observed a zero correlation. However, this meta-analysis integrated research findings from Western cultures. One explanation for our divergent results may be that the achievement motivations of Chinese adolescents differ from those of Western students [214,215]. Chinese youths “study not only for themselves but primarily for the status and prestige of their families” [215] (p. 135) and are under strong parental pressure to perform well at school. They are therefore mainly extrinsically motivated. However, individual interest, by contrast, is mainly an intrinsic phenomenon [216,217,218,219,220,221].

The correlation pattern, confirmatory factor analyses, and regression analyses underscore that it makes sense to measure school subject-related self-constructs in a specific way, tied to individual school subjects. The three school subject-specific ASC scales were proven to be overwhelming winners in the race against the other self-scales in predicting subject-specific academic achievements. They were the only ones that had notably unique powers to account for school grade variances. Noteworthy are the different proportions of variances in school grades, which were accounted for via the ASCs. For English, this corresponded to *r* = 0.49; for mathematics, it corresponded to *r* = 0.40; and for Chinese, it corresponded to *r* = 0.22. These divergent coefficients underline the relevance of subject-specific analyses. For the sake of comparison, it should be noted that in unselected WIRED samples (drawn from populations that are white, educated, industrialized, rich, and democratic), correlations between IQ and academic performance in individual subjects are usually in the range from *r* ≈ 0.40 to *r* ≈ 0.60 [88,90,91,92,93,94,95,96,222].

The remaining four self-scales were deemed to be irrelevant for predicting academic achievement after controlling for the associated ASCs. Thus, if the self-variables used in this study are taken to predict school grades, the measurement of subject-tied CSN, NFC, POE, and COI seems to be redundant if one also measures their corresponding ASCs. The reverse is not true. Measuring these four self-variables by no means eliminates the need to measure the ASC, at least for Chinese adolescents. In other words, our study provides additional insights into which of the five self-constructs surveyed should be prioritized in educational and psychological counseling and research. Subject-specific ASCs appear to be the key non-cognitive predictors of academic achievement.

Every research project has its limitations, and so does the present study. Our results were based on the administered questionnaires and school grades. Different operationalizations of predictors (e.g., other self-questionnaires) and different criteria (e.g., standardized achievement tests instead of grades) could lead to different outcomes. Methodologically, all the predictors were measured via self-reports. This may have slightly increased their intercorrelations due to possible common method variance (self-report bias) [223,224], but it did not affect their associations with school grades as the predictors and criteria were obtained from different sources.

Future research could use complementary alternative methods to assess the variables under study, for example, ratings by peers, teachers, and parents. Furthermore, since (achievement related) self-constructs develop and differentiate over the course of schooling, it is unknown whether these results are even valid for younger students. In addition, the sample consisted of Chinese adolescents. Studies comparing students from individualistic (Western) and collectivistic (Eastern) countries could explore whether and to what extent subjects’ cultural background is a relevant moderator variable in predicting academic achievement.

The present results are limited to two subjects from the verbal domain (Chinese as the native language; English as the first foreign language) and one from the numerical domain (mathematics). Future studies should also include other majors, such as science and history, as well as minors, such as music, sports, and arts.

The present study is a cross-sectional one. Research has repeatedly shown that ASCs and scholastic achievements are reciprocally connected [28,29,30,31,32,33]. It cannot be ruled out that this also applies to the other self-constructs that were measured. Longitudinal studies could reveal developmental changes in their complex nomological network and its relationship with academic achievement.

As with all correlational studies, causal conclusions are not possible. Randomized controlled training experiments are needed to figure out whether a training-induced improvement in one self-variable leads to an increase in other self-variables on the one hand and to an improvement in the associated academic performance on the other hand.

In summary, school subject-linked ASCs very much outperformed the school subject-linked self-variables CSN, NFC, POE, and CO in terms of statistically predicting assigned grades in two verbal subjects (Chinese and English) and in mathematics, at least among Chinese adolescents. Thus, ASCs are non-cognitive key predictors of academic achievement. For other purposes, i.e., beyond the performance predictions, CSN, NFC, POE, and COI may nonetheless be of great importance both in and out of school. Last but not least, assiduousness and diligence, desire for intellectual activities, willingness to learn continuously, and cultivation of individual interests should be valued as educational and psychological objectives and outcomes in their own right.

## Figures and Tables

**Table 1:** Factorial structures (three subject factors within each of the five self-related variables).

	χ^2^ (df)	CFI	SRMR	RMSEA [90% CI]	AIC
Academic self-concept (ASC)					
(18 items, 6 per subject)					
General factor	5567.58 (135)	0.35	0.19	0.23 [0.22, 0.23]	5675
Three subject factors:					
Chinese, mathematics, and English	397.83 (114)	0.97	0.06	0.06 [0.05, 0.06]	547
Conscientiousness (CSN)					
(24 items, 8 per subject)					
General factor	7750.12 (252)	0.38	0.13	0.19 [0.18, 0.19]	7594
Three subject factors:					
Chinese, mathematics, and English	472.28 (225)	0.98	0.05	0.04 [0.03, 0.04]	670
Need for cognition (NFC)					
(24 items, 8 per subject)					
General factor	6938.08 (252)	0.39	0.14	0.18 [0.18, 0.19]	7082
Three subject factors:					
Chinese, mathematics, and English	516.81 (225)	0.97	0.05	0.05 [0.04, 0.05]	714
Perseverance of effort (POE)					
(12 items, 4 per subject)					
General factor	2186.30 (54)	0.53	0.13	0.22 [0.21, 0.23]	2258
Three subject factors:					
Chinese, mathematics, and English	109.01 (39)	0.99	0.04	0.05 [0.04, 0.06]	212
Consistency of interest (COI)					
(12 items, 4 per subject)					
General factor	2129,49 (54)	0.53	0.13	0.22 [0.21, 0.23]	2201
Three subject factors:					
Chinese, mathematics, and English	75.45 (39)	0.99	0.03	0.03 [0.02, 0.05]	177

Notes. *N* = 791. All χ^2^: *p* < 0.001. Models with a general factor: maximal χ^2^/df = 41.24 (ASC); and minimal χ^2^/df = 27.53 (NFC). Models with three subject factors: maximal χ^2^/df = 3.49 (ASC); and minimal χ^2^/df = 1.93 (COI).

**Table 2:** Factorial structures (comprising five self-variables within each of three school subjects).

	χ^2^ (df)	CFI	SRMR	RMSEA [90% CI]	AIC
Subject: Chinese					
General factor	2326.85 (405)	0.78	0.07	0.08 [0.07, 0.09]	2507
Five factors: ASC, CSN, NFC, PER, and COI	1254.36 (395)	0.90	0.05	0.05 [0.05, 0.06]	1454
Subject: Mathematics					
General factor	2356.23 (405)	0.79	0.06	0.08 [0.06, 0.08]	2545
Five factors: ASC, CSN, NFC, PER, and COI	1236.28 (395)	0.91	0.05	0.05 [0.05, 0.06]	1483
Subject: English					
General factor	2322.57 (405)	0.81	0.06	0.08 [0.07, 0.08]	2503
Five factors: ASC, CSN, NFC, PER, and COI	1377.68 (395)	0.90	0.05	0.06 [0.05, 0.06]	1578

Notes. *N* = 791. ASC = academic self-concept (6 items), CSN = conscientiousness (8 items), NFC = need for cognition (8 items), PER = perseverance of effort (4 items), and COI = consistency of interest (4 items). All χ^2^: *p* < 0.001. Models with a general factor: maximal χ^2^/df = 5.82 (mathematics); and minimal χ^2^/df = 5.73 (English). Models with three subject factors: maximal χ^2^/df = 3.49 (English); and minimal χ^2^/df = 3.13 (mathematics).

**Table 3:** Psychometric properties of five subject-specific self-scales (school subjects: Chinese, mathematics, and English).

	Min. Score ^a^	Max. Score ^a^	M ^a^	SD ^a^	Mean *r*_it_ ^b^	Skewness ^b^	Kurtosis ^b^	α ^b^
Academic self-concept ^c^								
Chinese	6	36	19.35	5.97	0.64	0.24	−0.34	0.84
Mathematics	6	36	17.99	7.00	0.69	0.48	−0.40	0.88
English	6	38	17.61	7.16	0.69	0.30	−0.59	0.88
Conscientiousness ^d^								
Chinese	8	48	30.47	7.23	0.53	−0.07	−0.31	0.80
Mathematics	8	48	30.66	7.98	0.52	−0.11	−0.51	0.80
English	8	48	28.64	8.36	0.55	−0.17	−0.44	0.82
Need for cognition ^d^								
Chinese	8	48	30.62	7.58	0.57	−0.18	−0.24	0.84
Mathematics	8	48	31.44	8.34	0.58	−0.15	−0.47	0.84
English	8	48	28.38	8.72	0.60	−0.09	−0.50	0.85
Perseverance of effort ^e^								
Chinese	4	24	14.88	3.92	0.51	0.07	−0.34	0.71
Mathematics	4	24	14.66	4.53	0.52	−0.05	−0.61	0.73
English	4	24	13.76	4.56	0.53	0.07	−0.59	0.73
Consistency of interest ^e^								
Chinese	4	24	14.30	4.26	0.45	−0.10	−0.30	0.66
Mathematics	4	24	14.32	4.39	0.41	−0.04	−0.46	0.62
English	4	24	14.11	4.34	0.36	−0.18	−0.41	0.58

Note. *N* = 791. *r*_it_ = item discrimination index. ^a^ Raw scores, ^b^ items class-wise class converted into *z*-scores, ^c^ six items per scale, ^d^ eight items per scale, and ^e^ four items per scale.

**Table 4:** Intercorrelations (only decimals are given) of age, sex, subject-specific grades, and subject-specific self-scales (school subjects: Chinese, mathematics, and English).

		1	2	3	4	5	6	7	8	9	10	11	12	13	14	15	16	17	18	19	20
1	Age	—	04	00	−01	−04	−01	00	−03	04	04	03	−01	01	−01	02	03	02	−01	−03	−02
2	Sex		—	15	−04	28	11	−16	17	07	−09	11	15	−11	20	08	−08	12	03	−06	04
3	Grade-C			—	15	20	24	−04	04	13	−00	02	15	−04	02	14	−00	02	02	−03	−03
4	Grade-M				—	12	−00	40	−01	−02	25	−01	−04	28	−04	01	28	00	00	13	−01
5	Grade-E					—	05	−11	53	−04	−13	30	00	−13	37	−02	−11	27	03	−04	14
6	ASC-C						—	28	34	53	21	25	66	18	28	59	22	27	18	08	10
7	ASC-M							—	16	16	59	11	20	70	12	24	64	14	08	27	06
8	ASC-E								—	16	06	61	20	02	72	23	08	63	07	03	24
9	CSN-C									—	62	62	68	28	35	76	44	45	24	18	19
10	CSN-M										—	50	36	67	24	49	77	34	15	29	13
11	CSN-E											—	36	17	72	48	34	78	14	15	28
12	NFC-C												—	46	51	68	35	33	20	12	15
13	NFC-M													—	30	32	69	19	07	23	04
14	NFC-E														—	38	24	73	09	08	27
15	POE-C															—	58	58	24	21	24
16	POE-M																—	42	14	32	14
17	POE-E																	—	13	15	28
18	COI-C																		—	73	69
19	COI-M																			—	68
20	COI-E																				—

Notes. *N* = 791 (correlations with grades: *N* = 779); scoring of sex: 0 = male and 1 = female. C = Chinese, E = English, and M = mathematics; ASC = academic self-concept, CSN = conscientiousness, COI = consistency of interest, NFC = need for cognition, and POE = perseverance of E = effort. ǀ*r*ǀ > 09: *p* < 0.01 and ǀ*r*ǀ > 12: *p* < 0.001 (two tailed).

**Table 5:** Hierarchical linear regression analyses. Statistical prediction of subject-specific academic achievements (grades in Chinese, mathematics, and English) by sex and age (model 1), by sex, age, and subject-specific academic self-concept (model 2), and by sex, age, subject-specific academic self-concept, subject-specific need for cognition, subject-specific perseverance of effort, and subject-specific consistency of interest (model 3).

	Chinese Grade					Mathematics Grade					English Grade				
Model 1	β	*P*	*R* ^2^	∆*R*^2^	*p*-Value	β	*p*	*R* ^2^	∆*R*^2^	*p*-Value	β	*p*-Value		∆*R*^2^	*p*-Value
Sex	0.15	<0.001				−0.04	0.283				0.28	<0.001			
Age	−0.01	0.761				−0.01	0.862				−0.03	0.434			
			0.02	0.02	<0.001			<0.01	<0.01	0.556			0.08	0.08	<0.001
Model 2															
Sex	0.13	<0.001				0.03	0.402				0.20	<0.001			
Age	−0.01	0.772				−0.01	0.783				−0.02	0.529			
ASC	0.22	<0.001				0.40	<0.001				0.50	<0.001			
			0.07	0.05	<0.001			0.16	0.16	<0.001			0.32	0.24	<0.001
Model 3															
Sex	0.13	<0.001				0.03	0.413				0.20	<0.001			
Age	−0.01	0.731				−0.01	0.777				−0.02	0.619			
ASC	0.24	<0.001				0.38	<0.001				0.56	<0.001			
CSN	0.03	0.622				0.01	0.878				0.06	0.253			
NFC	−0.04	0.480				−0.02	0.687				−0.03	0.601			
POE	0.01	0.912				0.04	0.528				−0.13	0.011			
COI	−0.03	0.373				0.02	0.583				0.02	0.479			
			0.07	<0.01	0.854			0.16	<0.01				0.33	0.01	0.060

Notes. *N* = 779. ASC = academic self-concept, CSN = conscientiousness, NFC = need for cognition, POE = perseverance of effort, and COI = consistency of interest.

## Data Availability

Restrictions apply to the availability of these data. The data are only available upon reasonable request to the authors.
